# Supplementary material for: Multi‐dataset identification of innovative feature genes and molecular mechanisms in keratoconus
Source: J Cell Mol Med. 2024 Sep 19;28(18):e70079. doi: 10.1111/jcmm.70079 (PMC11412914; doi:10.1111/jcmm.70079)
Supplement: Supplementary file 2 — Material S2. [file JCMM-28-e70079-s002.zip › 7.GSEA analysis/3.DBNDD1/KEGG_DRUG_METABOLISM_OTHER_ENZYMES.html]

Details for gene set KEGG\_DRUG\_METABOLISM\_OTHER\_ENZYMES[GSEA]

|  || Dataset | DBNDD1.group.cls#h\_versus\_l |
| Phenotype | group.cls#h\_versus\_l |
| Upregulated in class | h |
| GeneSet | KEGG\_DRUG\_METABOLISM\_OTHER\_ENZYMES |
| Enrichment Score (ES) | 0.23318358 |
| Normalized Enrichment Score (NES) | 0.7441441 |
| Nominal p-value | 0.88842976 |
| FDR q-value | 1.0 |
| FWER p-Value | 1.0 |
Table: GSEA Results Summary

  

Fig 1: Enrichment plot: KEGG\_DRUG\_METABOLISM\_OTHER\_ENZYMES      
 Profile of the Running ES Score & Positions of GeneSet Members on the Rank Ordered List

  

| SYMBOL | TITLE | RANK IN GENE LIST | RANK METRIC SCORE | RUNNING ES | CORE ENRICHMENT || 1 | UGT1A8 | na | 313 | 0.502 | 0.1039 | Yes |
| 2 | UGT2B11 | na | 1393 | 0.345 | 0.1503 | Yes |
| 3 | UGT2B4 | na | 2640 | 0.266 | 0.1742 | Yes |
| 4 | UGT1A6 | na | 2660 | 0.265 | 0.2332 | Yes |
| 5 | UGT2B28 | na | 6027 | 0.150 | 0.1698 | No |
| 6 | CES2 | na | 8098 | 0.102 | 0.1329 | No |
| 7 | NAT2 | na | 8288 | 0.099 | 0.1496 | No |
| 8 | CYP3A4 | na | 8583 | 0.093 | 0.1620 | No |
| 9 | DPYS | na | 9622 | 0.077 | 0.1493 | No |
| 10 | ITPA | na | 12441 | 0.043 | 0.0777 | No |
| 11 | NAT1 | na | 12630 | 0.042 | 0.0816 | No |
| 12 | UGT2A3 | na | 13213 | 0.036 | 0.0730 | No |
| 13 | TYMP | na | 14289 | 0.028 | 0.0483 | No |
| 14 | UCK1 | na | 14529 | 0.026 | 0.0473 | No |
| 15 | IMPDH1 | na | 15303 | 0.021 | 0.0296 | No |
| 16 | CYP2A7 | na | 15639 | 0.018 | 0.0241 | No |
| 17 | UCKL1 | na | 16671 | 0.011 | -0.0031 | No |
| 18 | XDH | na | 16875 | 0.010 | -0.0066 | No |
| 19 | UGT2B10 | na | 17872 | 0.004 | -0.0345 | No |
| 20 | UMPS | na | 18159 | 0.002 | -0.0424 | No |
| 21 | UGT2B15 | na | 18962 | -0.004 | -0.0646 | No |
| 22 | CYP2A13 | na | 19140 | -0.006 | -0.0684 | No |
| 23 | UPP2 | na | 19460 | -0.008 | -0.0758 | No |
| 24 | TPMT | na | 19603 | -0.010 | -0.0778 | No |
| 25 | GUSB | na | 20109 | -0.014 | -0.0892 | No |
| 26 | IMPDH2 | na | 21792 | -0.030 | -0.1309 | No |
| 27 | HPRT1 | na | 22645 | -0.039 | -0.1468 | No |
| 28 | TK1 | na | 23404 | -0.048 | -0.1578 | No |
| 29 | DPYD | na | 23732 | -0.053 | -0.1554 | No |
| 30 | TK2 | na | 24274 | -0.060 | -0.1575 | No |
| 31 | GMPS | na | 24323 | -0.061 | -0.1452 | No |
| 32 | UGT2B7 | na | 24690 | -0.066 | -0.1409 | No |
| 33 | CYP3A7 | na | 26069 | -0.089 | -0.1607 | No |
| 34 | UCK2 | na | 26421 | -0.096 | -0.1492 | No |
| 35 | UGT2A1 | na | 28049 | -0.132 | -0.1664 | No |
| 36 | UPB1 | na | 28103 | -0.133 | -0.1380 | No |
| 37 | CES5A | na | 28155 | -0.134 | -0.1092 | No |
| 38 | UPP1 | na | 28283 | -0.137 | -0.0820 | No |
| 39 | CYP3A43 | na | 31748 | -0.251 | -0.1254 | No |
| 40 | CYP3A5 | na | 31787 | -0.253 | -0.0696 | No |
| 41 | CDA | na | 33080 | -0.334 | -0.0317 | No |
| 42 | CES1 | na | 33296 | -0.352 | 0.0413 | No |
Table: GSEA details [plain text format]

  

Fig 2: KEGG\_DRUG\_METABOLISM\_OTHER\_ENZYMES      
 Blue-Pink O' Gram in the Space of the Analyzed GeneSet

  

Fig 3: KEGG\_DRUG\_METABOLISM\_OTHER\_ENZYMES: Random ES distribution      
 Gene set null distribution of ES for **KEGG\_DRUG\_METABOLISM\_OTHER\_ENZYMES**
